# The Russian Doll Model: How Bacteria Shape Successful and Sustainable Inter-Kingdom Relationships

**DOI:** 10.3389/fmicb.2020.573759

**Published:** 2020-10-20

**Authors:** Enrica Pessione

**Affiliations:** Department of Life Sciences and Systems Biology, School of Nature Sciences, Università degli Studi di Torino, Turin, Italy

**Keywords:** phage–bacteria–human host, multitasking signals, evolution, partnership agreement, viral-like particles, moonlight proteins, PTMs (post-translational modifications), MVs (membrane vesicles)

## Abstract

Successful inter-kingdom relationships are based upon a dynamic balance between defense and cooperation. A certain degree of competition is necessary to guarantee life spread and development. On the other hand, cooperation is a powerful tool to ensure a long lasting adaptation to changing environmental conditions and to support evolution to a higher level of complexity. Bacteria can interact with their (true or potential) parasites (i.e., phages) and with their multicellular hosts. In these model interactions, bacteria learnt how to cope with their inner and outer host, transforming dangerous signals into opportunities and modulating responses in order to achieve an agreement that is beneficial for the overall participants, thus giving rise to a more complex “organism” or ecosystem. In this review, particular attention will be addressed to underline the minimal energy expenditure required for these successful interactions [e.g., moonlighting proteins, post-translational modifications (PTMs), and multitasking signals] and the systemic vision of these processes and ways of life in which the system proves to be more than the sum of the single components. Using an inside-out perspective, I will examine the possibility of multilevel interactions, in which viruses help bacteria to cope with the animal host and bacteria support the human immune system to counteract viral infection in a circular vision. In this sophisticated network, bacteria represent the precious link that insures system stability with relative low energy expenditure.

## Introduction

The existence of symbiotic relationships between bacteria and organisms belonging to other kingdoms has long been recognized, although only recently, thanks to the advancement of molecular and *omic* techniques, in-depth insights into their reciprocal interaction at a molecular level have been achieved (Qin et al., 2010; Mendes and Raaijmakers, 2015). The fundamental role that bacteria have played during higher organisms’ evolution, and the huge impact that they continue to have in host development, self-recognition, physiology and adaptation to changing environments have been extensively discussed (Zilber-Rosenberg and Rosenberg, 2008; Schnorr, 2015). Paradigmatic for describing these interactions are the examples concerning root (Ramírez-Puebla et al., 2013) and the animal gut (Ley et al., 2008) microbiota where both specificity and biodiversity have supported the establishment and evolution of what is now called holobiont, the unit on which evolutionary selection acts (Rosenberg and Zilber-Rosenberg, 2016).

In parallel to this multicellular organism-targeted relationship, one of the oldest and most important challenges for bacteria is the interaction with bacteriophages, viruses infecting prokaryotic cells. At a first sight, phages are parasites/predators that, after exploiting host biosynthetic machinery, kill the cell. From this standpoint, they represent a danger to be absolutely avoided by bacteria that have set-up complex mechanisms of phage resistance (Mirzaei and Maurice, 2017) that have in turn generated mutations in bacteriophages both to overcome resistance and to adapt to new bacterial species (Mirzaei and Maurice, 2017). The latter phage strategy promotes “host jumps” that provide a host-range expansion and ensures virus survival (De Sordi et al., 2019a). However, not only bacteria provide bacteriophage diversification, but also phages support bacterial diversification as shown by the high variability of phage receptors in the same bacterial species in a certain ecological niche (De Sordi et al., 2019b). These reciprocal dynamics, by creating a high selective pressure, have deeply contributed to the evolution of both partners resulting in the overall expansion of their genetic diversity, as suggested by Allen and Abedon (2013). This co-evolution has been recently confirmed to be very fast (just 8 days) by Wandro (2019) who observed that the interaction between Efv-phi1 lytic phage and its host *Enterococcus faecium* brought to mutations in the surface bacterial structures and RNA polymerase as well as in the tail proteins of the virus. Beside these surface modifications, bacteria can employ sophisticated strategies aimed to escape phage infection after nucleic acid injection. Among these, are worth mentioning the R-M system and the CRISPR (cluster regularly interspaced short palindromic repeats) sequences. The former involves restriction modification systems providing degradation of the phage genome (De Sordi et al., 2019b). The latter is consistent with abundant phage spacers that are identical to a sequence named the proto-spacer found in the genome of the infecting virulent phage. Therefore, this system counteracts nucleic acid invaders (both DNA and RNA) via a sequence specific strategy (Deveau et al., 2010). However, even in this case resistance mechanisms set up by viruses render both these mechanisms uneffective (Samson et al., 2013). In the case of CRISPR-based bacterial phage resistance, phage mutants exist that carry a single point mutation or a deletion in their proto-spacer and therefore they can successfully complete their lytic cycles (Labrie et al., 2010). The CRISPR system and the phage adaptive response represent one the best examples of co-evolution between bacteria and phages that shape the structure of microbial communities (Andersson and Banfield, 2008). On the other hand, bacteria have learnt how to cope with phage infection renegotiating this stressing event to arrange benefits for both contenders, and how to adapt to mammalian host immune system. This ability to convert a negative affair into an opportunity is part of the strategies referred in 2001 by Hoagland et al. (2001) that described the 16th rules of the living, among which three are worth mentioning: a) different assembling of simple modules to create diversity at low-cost, b) high information exchange to optimize instead that maximize, c) interconnection and integration of new aspects to change a bad event into an opportunity (i.e., changing predation/parasitism into symbiosis) These aspects are particularly relevant in the fast-growing and fast-evolving bacterial populations, however, they constitute a model also for guiding human behavior in a period (climate change and scarcity of resources) that reveals our overall fragility.

When considering bacterial interactions with both host and phage our traditional learning approach seems sometimes inadequate to fully understand the reality. The huge number of interactions and feed-backs, the difficulty to establish a clear cause/effect model, and the overall complexity of the network suggest that a circular and systemic point of view is best fitting for elucidating the whole dynamic. In the present review, I will try to explore, although partly in a reductionist way, the molecular mechanisms underlying this Russian doll model of multilevel interactions, considering on one side the molecular effectors at the host-bacteria interface and on the other side the bacterial advantages linked to phage infection. Finally, some aspects of the complex, systemic and holistic ecosystem made up by host, bacteria and phages will be discussed.

## The Interactive Biochemistry at the Bacteria Animal Host Interface

Bacteria–host interaction is an unpredictable relationship. What is clear analyzing the literature is that in a symbiosis the whole is more than the sum of the parts and therefore we cannot advance without a systems biology perspective (Relman, 2008). Often, the relationship becomes so intimate that both partners lose some genes and become strongly dependent on the presence of the other partner like in the inter-kingdom syntrophy [for example, lactic acid bacteria (LAB) that cannot synthesize amino acids and need milk proteins or animal inability to synthesize vitamins that are provided by the gut bacteria] (Ellers et al., 2012). This co-evolution has been in some cases so drastic that bacteria lost their identity to become organelles (Margulis’ theory) giving origin to a system of higher complexity (Dyall et al., 2004). Therefore, the apparent involution of each partner is sometimes the door to have access to a new life experience.

In humans, the presence of a commensal microbiota increases the host fitness providing new genes, thus conferring new metabolic capabilities, higher flexibility and adaptation to new ecosystems, like a pen-drive that enhances the capacity of a computer without dramatically altering the hard disk (Webster, 2014). However, besides genetics, several host factors (age, circadian rhythms, hormones, diet, and drugs) can affect symbiont bacteria diversity and single cell level phenotypic pattern (Zdziarski et al., 2010), therefore, the relationship should be considered dynamic and often unpredictable. This is the major challenge when exogenous microbiota supply is planned to treat gut dysbiosis (De Vrieze, 2013) or enhance plant fitness using eco-friendly agriculture (Berg et al., 2016). In this scenario, molecular communication and co-metabolism are crucial factors in host-microbe interactions.

Cross-signaling between bacteria and their host is a well-established concern (Mazzoli and Pessione, 2016). From one side, the host can modulate microbiota releasing compounds (top-down control) and, on the other side, bacteria can use their signaling molecules to modify host biology (bottom-up control) also responding to host stimuli (Cryan and Dinan, 2012). Actually, it has been experimentally proven that this reciprocal interaction modifies the gene expression profiles of both partners (Zdziarski et al., 2010). Host-derived factors besides modulating microbiota composition (Hevia et al., 2015) can impact bacterial genomics, transcriptomic and proteomics at a single cell level, altering the virulence profiles (Denou et al., 2008; Sandrini et al., 2014) and thus conditioning microbial evolution and diversification. As an example the expression of virulence genes is often elicited by host factors such as stress-induced catecholamines (Cogan et al., 2007) toward whom bacteria have evolved specific receptors (Clarke et al., 2006). Compounds of exogenous origin such as antibiotics should be included among modulating host-supplied factors. Actually, low-levels of antibiotics (like those acquired from the diet) can induce expression of virulence genes and favor DNA exchanges between bacteria (Goh et al., 2002). In parallel, microbial factors, both surface-bound and secreted, influence host physiology by regulating several functions as better described in the next section (Liu et al., 2020). Although secondary metabolites are specifically produced by microbes to interact with their environment and therefore with organisms sharing the same ecological niche (social adaptation) (O’Brien and Wright, 2011), frequently simple molecules derived from central carbon catabolism also play a role in cross-communication.

### Bacterial-Derived Multitasking Signaling Compounds

Several bacterial-produced metabolites deeply affect the overall host physiological status, including metabolism (Bäckhed et al., 2004), immunity (Segain et al., 2000), and mental health (Cryan and Dinan, 2012). The three main families of biochemical compounds (lipids, sugars, and proteins) all contribute to bacteria signaling toward the host. Their multitasking roles, targeting bacteria and host, are reported in Table 1.

**TABLE 1:** Multitasking bacterial-derived compounds and their different roles in bacteria and host.

**Compounds**	**Function for bacteria**	**Function on host**	**References**
SCFA	Catabolism end-products	Histone deacetylase inhibitors Control of tight-junction proteins Anti-inflammatory action (increased IL-10, TGF-β, and annexin A1)	Davie (2003), Wang et al. (2018), Vieira et al. (2017)
EPS	Osmoprotectants Biofilm polymeric matrix	Inhibit the growth of Caco-2 colon cancer cells Decrease the production of TNF-α and increase the IL-10 production Innate and adaptive immune responses	Deepak et al. (2016), Sungur et al. (2017), Hidalgo-Cantabrana et al. (2014)
Amino acid derivatives Histamine, serotonin, and nor-epinephrine 3-oxo-C12 homoserine lactone GABA	Alkalinization and energy gain QS autoinducers	Control enteric neurotransmission Modulate pro- and anti-inflammatory cytokine ratio Modulation of neuro-muscular junctions and blood pressure	Mazzoli and Pessione (2016), Glucksam-Galnoy et al. (2013), Mazzoli et al. (2010)
Peptides	QS, bacteriocins	Alter cytokine profiles of both monocytes and dendritic cells	Meijerink et al. (2010), Van Hemert et al. (2010)
Moonlight proteins	Glycolisis, TCA, and stress chaperones (GroEL and DnaK)	Tissue adhesion Dendritic cell maturation Enhance secretion of IL-8 in human macrophages Induce apoptosis in gastric cells	Granato et al. (2004), Floto et al. (2006), Bergonzelli et al. (2006), Basak et al. (2005)

*EPS, exopolysaccharides; GABA, γ-amino butyric acid; IL, interleukin; QS, quorum sensing; SCFA, short chain fatty acids; TCA, tricarboxylic acid cycle; TGF-β, tumor growth factor β; TNF-α, tumor necrosis factor α.*

Short-chain fatty acids (SCFA), namely acetate, lactate, propionate and butyrate, are generally end-products of bacterial carbohydrate catabolism, mainly produced following a fiber-rich diet. By acting as histone deacetylase inhibitors they can induce epigenetic modifications that modulate host functions such as energy storage and immune homeostasis (Davie, 2003). Among SCFA, acetate displays an interesting role in controlling articular diseases by inducing faster resolution of the neutrophil-mediate inflammatory response (Vieira et al., 2017). Butyrate (in concentrations lower than 2 mM) can improve the gut epithelial barrier by enhancing the production and secretion of mucin by goblet cells (Burger-van Paassen et al., 2009) and the expression of tight-junction proteins (Wang et al., 2018) besides stimulating the production of anti-inflammatory cytokines, such as interleukin (IL)-10 (Vipperla and O’keefe, 2012). Butyrate also helps to regulate the balance between proliferation, differentiation, and apoptosis of colonocytes and it can be found in higher quantities in the feces of healthy individuals compared with individuals with colorectal cancer (Serban, 2014).

Exopolysaccharides (EPS), besides being important constituents of the biofilm extracellular polymeric substance and acting as protective shield for preventing cell desiccation and osmotic stress (Pessione, 2012) also have a role as signaling molecules, behaving as growth control agents and immune modulators for the host. *Lactobacillus acidophilus* EPS can inhibit the growth of Caco-2 colon cancer cells (Deepak et al., 2016) while EPS from *L. gasseri* can induce apoptosis in cervical tumor cells, also decreasing the production of TNF-α and increasing the IL-10 production thus controlling inflammation (Sungur et al., 2017). Also *Bifidobacteria*-derived EPS support important functions such as modulation of the composition of the gut microbiota and of communication processes with the host. In this genus the G + C content of the genes encoding EPS synthetic enzymes is different from the one of the whole genome suggesting horizontal gene transfer (HGT) events that underline the importance of acquiring such capability (Hidalgo-Cantabrana et al., 2014).

However, the central role is played by nitrogen compounds such as amino acid (and their metabolic products) peptides and proteins. Glutamate, glutamine, and their derivative gamma aminobutyric acid (GABA), especially produced by LAB, are involved in neuromodulation and in controlling neuro-muscular junctions as well as vascular tension and blood pressure (Mazzoli et al., 2010). The tryptophan-derivative indole can control tight junctions (Bansal et al., 2010). Other amino acids derivatives such as histamine, serotonin, nor-epinephrine as well as inorganic gaseous molecules such as NO and H_2_S (generated by bacterial conversion of diet compounds) are responsible for controlling enteric neurotransmission (for exhaustive reading, see Mazzoli and Pessione, 2016).

It is interesting to underline that bacteria produce SCFA and EPS for their own benefit and also amines have a specific function in both pH buffering and energy gain increase for the prokaryote partner (Pessione, 2012). Therefore, this host-targeted effect seems to be an additional and cheap function of an otherwise useful molecule.

A further example concerns quorum sensing (QS) autoinducers. In *Pseudomonas* 3-oxo-C12 homoserine lactone can control different host metabolic pathways including tight-junction proteins modifications, resulting in an altered gut barrier (Vikström et al., 2010), pro- and anti-inflammatory cytokine ratio (Glucksam-Galnoy et al., 2013) and apoptosis (Tateda et al., 2003). Similarly, *Pseudomonas* quinolone signal can downregulate innate immune responses (Kim et al., 2010) and control in a concentration-dependent manner host neutrophil chemotaxis (Hänsch et al., 2014). A review article by Dobson et al. (2012) elegantly illustrates the multiple roles that Gram-positive secreted peptides can have: first, they behave as QS signals at low concentrations and as bacteriocins at higher concentrations by inducing their own biosynthesis when a cell density threshold is reached (Kleerebezem, 2004). Secondarily, at least in *L. plantarum*, they can behave as bacteriocins and immune modulating agents altering cytokine profiles of both monocytes (Van Hemert et al., 2010) and dendritic cells (Meijerink et al., 2010). All these examples underline the sustainability of these multitasking molecules that support energy saving for bacteria that use the same compound to achieve many different functions. It is evident that host cells must have evolved receptors for sensing such “non-self” compounds, but it is even smarter the fact that bacteria can use a single molecule to orchestrate relationships with both their siblings and their host, with low energy expenditure, and using the same “language.”

Alongside these small molecules, important mediators for bacteria–host interaction are surface-bound and secreted proteins. Surface-layer proteins and glycoproteins can control bacterial adhesion (Beganoviæ et al., 2011) and some secreted proteins like p40 and p75 can cooperate to the gut epithelium physiology by protecting tight junctions and enhancing IgA production (Wang et al., 2017). However, the emblematic example of a cheap bacterial strategy to fulfill a successful interaction with the animal host is the existence of moonlighting proteins (MPs).

### Moonlighting Proteins

Moonlighting proteins are proteins that perform different functions in different cellular compartments (Jeffery, 1999). Some exceptions concern MP having a double task in the same cellular compartment such as surface-linked proteases which also behave as adhesins (Kainulainen and Korhonen, 2014). They are ubiquitous in all living kingdoms and their existence has finally overcome the concept one gene-one function, thus providing a clear explanation for the low number of genes present in most living organisms’ genomes (Henderson and Martin, 2014). In bacteria, MPs represent a very interesting example of economy that exploits the same macromolecule to achieve housekeeping functions inside the cell and to interact with the host when secreted (Table 1). Most of them are central metabolism enzymes (especially glycolytic or TCA cycle enzymes) elongation factors (EF-Tu and Ef-G) and stress-induced proteins (chaperones such as GroEL and DNAk) that after secretion can interact with the animal host favoring bacterial adhesion to mucosal surfaces (when surface-bound) (Jeffery, 2018) and regulating the host immune system (when released) (Henderson et al., 2008). Although it has been recognized that these proteins use different domains to fulfill their intracellular and moonlighting roles, in some cases the two functions are performed by the same molecular region as described for the glyceraldehyde 3-phosphate dehydrogenase (GAPDH) whose adhesive function is inhibited by the co-factor NAD^+^ that hinders the catalytic site (Kinoshita et al., 2008).

Moonlighting proteins lack any classical export signal sequence related to the most common Sec or TAT secretion machineries and any surface attachment domain (Jeffery, 2018). Although the secretion mechanism for most MPs is not known, some possible non-canonical export ways have been described. Transient post-translational modifications (PTM) have been hypothesized to occur, acting as a signal for partitioning some proteins (among the pool of a certain metabolic enzyme) outside the cell. Enolase, a glycolytic enzyme, can be spontaneously modified by its substrate (2-phosphoglicerate) at the level of lysine_341_, thus becoming suitable for extracellular release. Catalytically active mutants of enolase bearing glutamine, glutamate, arginine, or alanine in the residue_341_, cannot be extruded (Boël et al., 2004). A second hypothesis suggests that secondary and tertiary structures could be important for recognition and secretion: in *Bacillus subtilis*, a hydrophobic alpha-helical domain within enolase that contributes to its secretion has been identified (Yang et al., 2011). Since secreted and intracellular MPs share the same physical structure, the existence of chaperones inducing transient rare conformations that are competent for secretion has been hypothesized but not yet proven (Amblee and Jeffery, 2015). Finally, bacterial membrane vesicles (MVs), better described in the next section, can be exploited to release MP (Henderson and Martin, 2014) as previously demonstrated for similar structures present in human cells (Lancaster and Febbraio, 2005).

Regarding surface binding, classical anchoring motifs are lacking in MPs. Some authors ascribe the capability to be retained on the cell surface to specific conformational states of each protein. It has been demonstrated that GroEL1 is prevalently released into the external environment (Cehovin et al., 2010) whereas GroEL2 is a capsule associated protein (Hickey et al., 2010) in Mycobacteria. While the latter favors adhesion to macrophages, the former is the best fitting in inducing cytokine release by monocytes (Lewthwaite et al., 2001). According to others, environmental factors such as pH, also play a role in the cell-anchoring or release of a specific proteins (Antikainen et al., 2007). Because of their different biological tasks, cell-anchored and released MPs both cooperate to achieve a coordinated interaction with the host. Curiously, the MPs released by a bacterial species can re-associate on the surface of a different one, suggesting that the communication occurs among more than two partners (Oliveira et al., 2012).

As far as adhesion is concerned, there is evidence that bacteria displaying surface-bound MPs can auto-aggregate (a pre-requisite for mucosal adhesion) (Waśko et al., 2014) and also co-aggregate with other bacterial species (a feature that potentially quenches pathogens) (Bergonzelli et al., 2006). Adhesion targets in the host are mucus components (Kinoshita et al., 2008), gut epithelial cells (Granato et al., 2004) or extracellular matrix proteins (collagen, laminin, and fibronectin) (Castaldo et al., 2009; Genovese et al., 2013). A peculiar type of interaction has been described between two MPs, the mammalian MP Hsp60 and the bacterial (*Listeria monocytogenes*) MP alcohol-acetaldheyde dehydrogenase, both acting inside the cell as cell stress proteins (Wampler et al., 2004). Actually, in multicellular organisms, membrane exposed MPs act as receptors for bacteria (Jin et al., 2003) and viruses (Reyes-del Valle et al., 2005).

Adhesion ability represents an advantage when microbes live in symbiosis with higher organisms, favoring tissues colonization. It is interesting to underline that, since pathogens and commensal bacteria share most of the moonlighting adhesins, beneficial bacteria can act as competitors for gut epithelium adhesion thus supporting surface exclusion of the pathogens (Servin, 2004). In LAB that are paradigmatic examples of probiotic organisms, MPs localize on lipoteichoic acids and in cell division sites with ionic interactions (Kainulainen and Korhonen, 2014). Their extracellular location (surface-attached or released into the environment) depends upon pH: under acidic conditions (pH lower than their isoelectric point, i.e., 5) some positively charged MPs (like enolase and GAPDH) can interact with the negatively charged lipoteichoic acids, whereas at higher pH the molecules, negatively charged, are released from the cell surface (Antikainen et al., 2007). These considerations, however, are not true for some pathogenic bacteria that retain the MPs cell-anchored even in neutral and alkaline conditions (Jagadeesan et al., 2010). Hence, since cell-wall anchored MPs act as adhesins, competitive displacement of pathogens on host mucosal surfaces by beneficial microbes like LAB can only occur in acidic conditions, underlining the huge importance of the environment in balancing commensal–pathogens–host interactions. In this respect extracellular and envelope proteomic data suggested that a selenium supply in the growth medium can enhance the abundance of some adhesive MPs (alpha-enolase, EF-Tu) in *L. reuteri* Lb2BMDSM16143 (Mangiapane et al., 2014).

Released MPs prevalently act as immune modulating factors. Immune modulation is especially elicited by moonlighting bacterial chaperones. i) GroEL/Hsp60 acts activating human monocytes (Maguire et al., 2002) and stimulates the secretion of IL-8 in human macrophages (Bergonzelli et al., 2006). ii) Bacterial intracellular survival into macrophages is provided by Hsp20 (Schnappinger et al., 2003). iii) A cell-stress protein peptidyl-prolyl isomerase (PPI) also promotes survival inside macrophages (Henderson and Martin, 2011). iv) DNAk stimulates dendritic cells maturation (Floto et al., 2006). Although the last three examples refer to pathogenic bacteria, nevertheless released MPs seem to play an important role in controlling prokaryote–host interaction at multiple levels.

Additional activities of secreted bacterial MPs include i) induction of apoptosis in gastric epithelial cells by PPI (Basak et al., 2005) and ii) the role of GroEL/Hsp60 from *Enterobacter aerogenes* as toxin available for the insect host to paralyze its prey (Yoshida et al., 2001). Finally, according to Henderson et al. (2008) we can conclude that among MPs, stress chaperones, having evolved specifically to interact with other proteins, may have cell signaling functions similar to cytokines, sometimes creating a network of interaction with host-released MPs.

The number of MPs identified by mass spectrometry (MS) and reported to be displayed on the bacterial cell surface is increasing every day (Wang and Jeffery, 2016). Interestingly, bacterial MPs besides interfacing with the mammalian host can also favor symbiotic relationships between bacteria and yeasts (Katakura et al., 2010) suggesting that they double role has evolved to promote whatever inter-kingdom interactions.

### PTMs and Cross-Talk

A further fascinating aspect of the bacteria–host cross-talk is the role of PTMs on proteins. At the end of the last century, once achieved exhaustive information about the majority of plant, animal and microbial genomes an unexpected low number of genes was found. If in Eukarya the presence of introns can partially account for this finding, in prokaryotes it cannot. An important step to elucidate this point is the huge presence of PTMs on proteins. Although for long time bacteria were considered unable to post-translationally modify proteins, it is now evident that the paradigm one gene-one protein is wrong even for prokaryotes (Cain et al., 2014).

**FIGURE 1:**
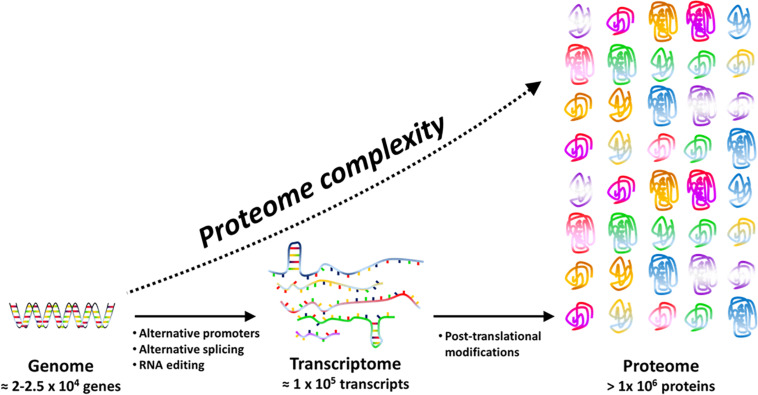
Proteome complexity is the result of transcriptional (e.g. alternative promoters), post-transcriptional (e.g. RNA editing and splicing) and post-translational events. Post-translational events include a large panel of reversible or irreversible chemical modifications that may affect structure, function and cellular location of proteins.

The concept of protein species (Jungblut et al., 2008), firstly introduced to avoid confusion with the term “isozyme” (that mean the product of a different gene encoding a protein with analogous function, e.g., catalyzing the same reaction with different efficacy and sometimes different direction), refers to different PT modified proteins and is now well accepted. There is an increasing evidence that PTMs have a huge biological significance because they determine peculiar protein properties like half-life, interaction with other proteins, cellular localization (Kobir et al., 2011) as well as diversified protein functions such as redox signaling, enzyme activity, chemotaxis, and metabolic flux tuning (Dykstra et al., 2013). Actually, each protein species (for instance a protein bearing glycosylation) can solve a specific physiological function not performed by the same gene product that has been processed differently (for instance the same protein when phosphorylated). As an example, methylations or acetylations of cellulosome scaffold proteins have critical functional significance, by impacting differently cellulose utilization efficiency (Dykstra et al., 2013). In this way, only one gene can support several tasks thus guarantee economy of genes and of energy required for transcription and translation (Figure 1).

An interesting concern of PTMs is their regulatory/interactive role. Cellular sensing, adjusting and responding to changes in the external environment, is often mediated by reversible PTMs and post-translational regulatory networks in which one PTM can compete with or, on the contrary, facilitate further PTMs by inducing conformational changes on the targeted protein (Soufi et al., 2012) (for comprehensive information on the evolution and functional significance of PTMs see the review by Beltrao et al., 2013). In the specific case of bacteria, the majority of PTMs are transient and occur to ensure rapid adaptation to changing conditions (Grangeasse et al., 2015). Furthermore, in the case of commensal/pathogenic bacteria PTMs on key extracellular proteins can support interaction with the host: serine-glycosylated proteins are important in bacterial adhesion and colonization of host mucosal tissues (Zhou and Wu, 2009), acetylated proteins play a role in virulence (Ren et al., 2017) and multiple PTMs are used by bacteria to alter their surface antigens thus escaping host immune response (Cain et al., 2014).

However, the most intriguing feature is the ability of bacteria to post-translationally modify host proteins (Ribet and Cossart, 2010). This can be achieved by either direct PTM of surface exposed host proteins or by interfering with the host PTM machinery by means of secreted molecules able to penetrate inside the host cell. The preferential targets of these bacterial PTMs are host regulatory proteins like Rho GTPases, since with a single modification on them, bacteria can activate or deactivate the control of a complex network of signals inside the host cell. For instance, RhoGTPases can be either activated by deamidation and/or polyamination or rather inactivated by glycosylation and/or AMPylation thus controlling crucial events such as innate and adaptive immune responses (Ribet and Cossart, 2010). Bacteria can also impact host gene transcription, as occurs by histone-targeted PTMs (Hamon and Cossart, 2008). It is evident that discriminating between “simple” PTMs and epigenetic effects when considering histones is artificial, so the conclusion that bacteria can deeply influence, by epigenetic effects, host gene expression is not so nonsense. Considering that other bacterial modifications on non-proteinaceous molecules have been reported (Pizarro-Cerdá and Cossart, 2004), also nucleic acid modification cannot be totally excluded. As an example, commensal gut bacteria can methylate the DNA of the TLR4 gene thus regulating intestinal inflammation and contributing to the maintenance of intestinal symbiosis (Takahashi et al., 2011) and several pathogens can induce histone acetylation and DNA methylation in the host during infection (Schmeck et al., 2005; Bierne et al., 2012). This opens a new area of investigation on the complex interaction network between bacteria and their hosts. Further information in this field, especially concerning the epigenetic modulation of host gene expression through PTM of histones exerted by commensal/symbiont bacteria, will provide added value to the knowledge of the mechanisms involved in bacteria–host reciprocal control and in supporting the theory of a superorganism constituting the holobiome selection unit (Rosenberg and Zilber-Rosenberg, 2016).

## The Inner Host: Bacteria-Phage Contract, a Hidden Opportunity

Bacteria–phage interaction can result in the well known lytic (phage replication and cell lysis) and lysogenic (phage integration into bacterial host DNA) cycles. Occasionally, in particular conditions also pseudolysogeny (phage enters and remains in the cytoplasm without integrating into the bacterial DNA and without replicating) (Cenens et al., 2013) and the so-called chronic cycle (phages particles are continuously released without causing cell lysis) (Mirzaei and Maurice, 2017), can occur. The former evolves toward lytic or lysogenic cycles whereas the latter can cause a lower growth rate in the host bacterial cell because part of the cell energy is directed to phage functions (Munson-McGee et al., 2018).

The phage lytic cycle involving bacterial killing is frequently observed in open environments (soils and waters) where the number of viral particles is about 50 fold higher than the number of living cells (Srinivasiah et al., 2008). Besides promoting antagonistic co-evolution (De Sordi et al., 2019a,b) the lytic cycle is responsible of nutrient cycling among bacterial populations, mineralization of organic bacterial matter and ultimately enrichment of soils (Allen and Abedon, 2013). However, lytic cycle is not the rule in well-established ecosystems such as the microbial biofilm (Jayaraman, 2008) and the human gut (Mirzaei and Maurice, 2017) where lysogenic events mediated by temperate bacteriophages are prevalent because of several mechanisms of resistance set up by bacteria against virulent viruses (Knowles et al., 2017). Independently from the final outcome (lytic, chronic, pseudolysogenic, or lysogenic cycle) and from the stress that phage attack causes to the bacterial cell, phage infection seems to have ensured during evolution some advantages to the bacterial populations.

### Possible Advantages Related to Phage Infection

**FIGURE 2:**
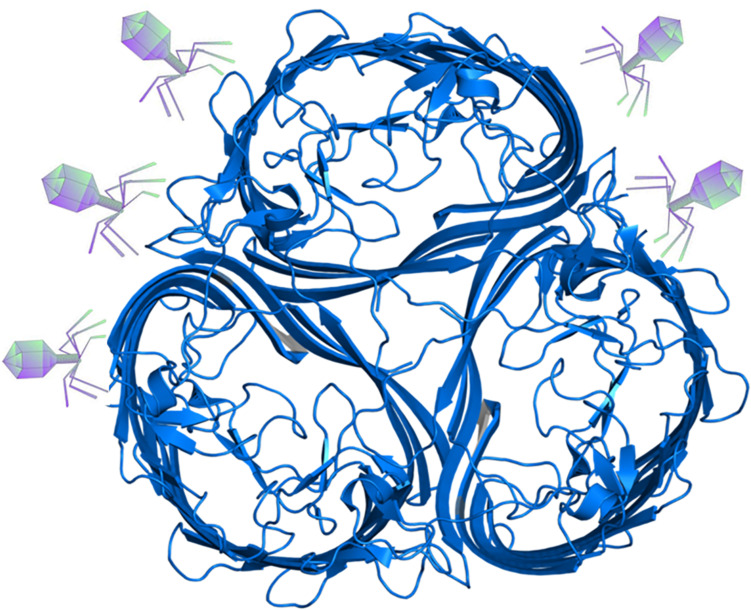
The maltoporin facilitating maltose uptake is a phage receptor.

Bacterial proteins that are involved in nutrient uptake (such as porins present on the outer membrane of Gram-negative bacteria), are often used by phages as receptors. In *Escherichia coli*, as an example, the receptor for the tails of the phages T5, TI, and phi-80 (the ton A/ton B proteins) is used for ferric iron (ferrichrome) uptake (Braun et al., 1976) and in *Salmonella* the T5-like phage receptor is the uptake protein for vitamin B_12_ (Kim and Ryu, 2011). In *E. coli*, it has long been established that the lambda-phage receptor (LamB protein) is a trimeric maltoporin which facilitates maltose uptake (Szmelcman and Hofnung, 1975), although the specific binding sites for phage and for the disaccharide are located on different amino acid residues on this protein (Charbit et al., 1988) (Figure 2). It seems therefore that the risk of phage entry is the price to be paid by the bacterial cell to gain food in nutrient-poor environments. Nevertheless, a question arises: why bacteria (whose ability to resist to phage attack with an arsenal of resistance mechanisms is well-known, see for example Mirzaei and Maurice, 2017) continue to harbor a protein that facilitates phage infection, without modifying it to render it suitable only for nutrient uptake? Evidently, the benefits of obtaining essential nutrients overwhelm the negative effects of phage attack, otherwise this system would be lost or modified. It is tempting to speculate that phage attack to the receptor can induce conformational changes on the protein so as to render it most effective in nutrient capturing. However, at present there are not experimental data supporting this hypothesis. Alternatively, these proteins could have been conserved since phage attack, because of the numerous benefits described in the next sections, cannot be seen only/always as a negative event.

**FIGURE 3:**
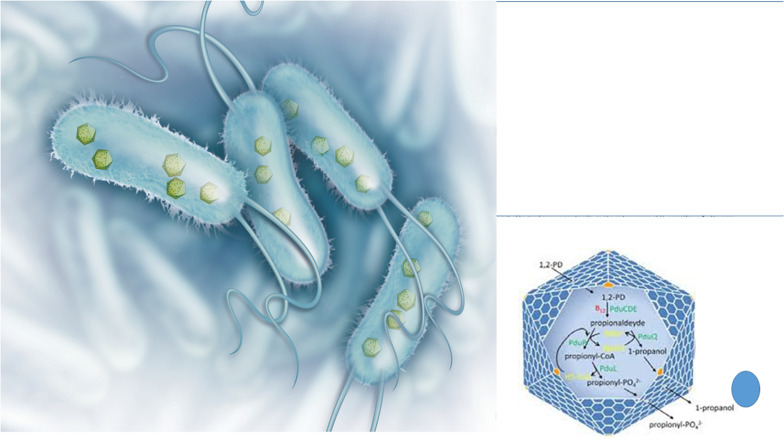
Bacterial structures showing similarities with phages: protein shell microcompartments and the phage head. Microcompartments are used for isolating metabolic pathways generating toxic compounds such as 3-hydroxypropionaldehyde (*on the left*: illustration originally printed in The Scientist by Thom Graves; *on the right*: from Lundin et al., 2020).

A peculiar phenomenon whose origin is still controversial is the presence of viral-like structures inside prokaryotes (Figure 3). These protein-based particles, similar to phage capsid (but lacking viral nucleic acid), represent a sort of a primitive compartimentalization strategy allowing the formation of micro-compartments or organelles in bacteria. These protein shells confer evolutionary advantage to the bacterial host because they isolate from the rest of the cytosol peculiar metabolic pathways generating toxic compounds such as aldehydes. It is well known that 3-hydroxypropionaldehyde (produced during the conversion of glycerol to 1,3-propanediol) is toxic for several Gram-negative bacteria such as *Klebsiella*, *Enterobacter*, and *Citrobacter* (Barbirato et al., 1998) and it is even used for food preservation being active also toward *Listeria monocytogenes* and *E. coli* O157:H7 (Vollenweider and Lacroix, 2004). In *L. reuteri* the 3-hydroxypropionaldehyde is named reuterin and, when suitably converted into acrolein, is used as bacteriocin-like weapon conferring ecological advantage to this bacterial species toward surrounding bacterial populations (Engels et al., 2016). In *Salmonella* the enzymes for the metabolization of 1,2 propanediol, generating toxic propionaldehyde are encapsulated in microcompartments (Sampson and Bobik, 2008) that in their aspect and architectural organization (polyhedral protein shells) are very similar to phage particles, especially phage-head. The possible viral origin of these particles is very likely, however, at present, only in Archea the structure (named encapsulin) reveals a common ancestry with the capsid protein of a virus (Sutter et al., 2008). On the other hand, the Gene Transfer Agents are viral-like particles from defective phages that harbor in the capsid bacterial DNA fragments that can be transferred to suitable recipient bacteria sustaining recombination (Lang et al., 2012). These particles do not contain phage nucleic acids, but the bacterial host harbors a prophage in its genome.

**FIGURE 4:**
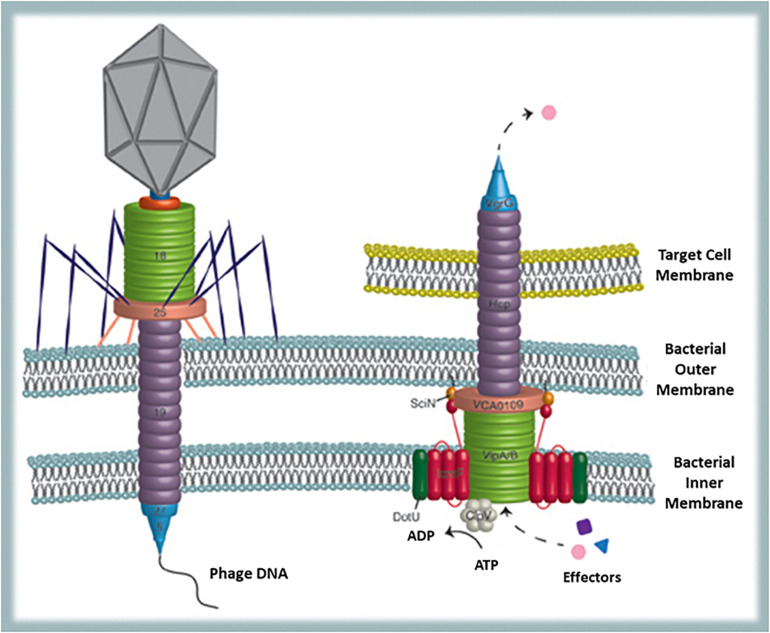
Bacterial structures showing similarities with phages: T6SS and the phage tail. T6SS is used to translocate completely folded proteins across inner and outer membrane of the secreting bacterium and across other bacteria’s or eukaryotic host membranes (from Records, 2011).

Finally, a possible viral remnant present in a certain number of Gram-negative bacteria is the type VI secretion system (T6SS). This is a phage-like structure very similar to phage T4 contractile nanomachine (Basler et al., 2012). Actually, T6SS can be illustrated as an inverted phage tail on the surface of the bacterial cell, used for secreting proteins in the extracellular environment or for injecting them directly into target (bacterial or host) cells (Records, 2011) (Figure 4). Structural homologies are common in biology and also the type II secretion system proved to be similar to filamentous phages (Leiman et al., 2009). Nevertheless, besides structural homologies, in the T6SS sequence similarities to phage T4 tail tube are also present (Leiman et al., 2009). Since diversification of functions from one original structural model can occur during evolution, establishing whether the phage tail preceeds the secretion system or *vice-versa* is hard matter. However, according to Records (2011) it is likely that some Gram-negative bacteria have utilized for their own benefit proteins encoded by phage genes integrated into their genomes. This secretion system offers to bacteria numerous advantages. Actually, among the seven known ways that bacteria evolved to export proteins (type I–VII secretion systems) T6SS is one of the most interesting: it provides a very efficient mean (several proteins can be delivered simultaneously into the target cell) to translocate completely folded proteins across inner and outer membrane of the secreting bacterium and also across other bacteria’s or eukaryotic host membranes (Basler and Mekalanos, 2012). Sometimes protein transfer is finalized to adjacent bacterial cell killing and acquisition of DNA (that can be used as nitrogen/phosphorus source but also for gene recombination) from the prey (Ringel et al., 2017). This strategy, although initially costly in ATP consumption, supports competition between bacteria as well as competence and HGT (Basler, 2015). Furthermore, proteins secreted by T6SS are exchanged among cells and reused for T6SS assembly thus providing recycling (sustainable and shared use of resources) and promoting cooperation between strains to kill competitors (successful interaction) (Vettiger and Basler, 2016). Furthermore, regarding bacteria–host interaction, it has been shown that mutant strains lacking a T6SS orthologous gene trigger stronger inflammatory responses than usual, suggesting that T6SS may function as modulator of acute inflammation thus promoting long-term interaction between bacteria and host (Chow and Mazmanian, 2010). Finally, since genes for T6SS are also conserved in free-living bacteria, it reasonable to assume that besides being involved in interbacteria and host interaction, T6SS also improves the environmental fitness of the bacteria possessing it.

### Lysogeny and Its Numerous Benefits

Lysogeny cannot be considered just as a simple harboring of “selfish DNA” since it offers some basic advantages to the infected bacterium in whatever habitat, such as the immunity toward further viral infections (Richter et al., 2012) and new functions related to stress resistance (Bondy-Denomy and Davidson, 2014). In specific conditions, other advantages (best fitness) are related to the ecological niche. In open aquatic environments, although the lytic cycle seems to be the general pattern (affecting periodical fluctuations in the bacterial population biodiversity) phage islands controlling genes involved in photosynthesis and light detection have been found in Cyanobacteria genome (Lindell et al., 2007). Ultimately, this results in a better photosynthetic fitness of the bacterial host.

However, it is in complex ecosystems (biofilms, animal-, and vegetal-associated microbiota) that lysogeny reveals its huge potential in driving evolution, suggesting multilevel effects on different partner cells. In the best-studied ecological niche, the human gut, most phages bear integrase genes allowing the prophage lifestyle (Minot et al., 2011). On the other hand, the transition lysogenic-lytic is sometimes dependent also upon QS autoinducers produced by the bacterial host (Silpe and Bassler, 2019). Temperate phages act as vectors for HGT (resulting from encapsidation of bacterial genes by a prophage when the environmental conditions favor a lytic cycles) frequently occurring when bacterial cells are in close proximity. The gene recombination that occurs in a certain ecosystem, favors the exchange of characters crucial for enhancing the metabolic and colonization capabilities of bacteria, meanwhile supporting population diversity and ultimately survival (Harrison and Brockhurst, 2017). However, a recent evaluation, based on a bioinformatics approach, excludes a real role of phages in the propagation of genes involved in antibiotic resistance: actually, the phage genome rarely encodes antibiotic resistance genes and transduction events, being based on erroneous encapsidation of non-viral DNA, seldom target these genes (Enault et al., 2017).

In parallel to the advantages of HGT, phage themselves act as a reservoir of genes important for the host fitness and especially for their social life and interkingdom relationships. Actually, integrated prophages, whose existence depends upon bacterial host survival, can alter virulence gene expression and generally account for a huge number of opportunities offered to their bacterial host (Wandro, 2019). The toxin-antitoxin modules (TA) present in the prokaryote genome have been described as originated from defective viral sources (Villarreal, 2012). They can encode several items. Among these, bacteriocins and immunity factors toward the self-produced bacteriocin (Tikhonenko et al., 1975) can offer advantage to a specific bacterial subpopulation in the fight against other bacteria. The bacteriocin producers therefore, soon become “permanent holder” of the viral genetic element conferring this benefit. Some TA genes such as mazEF induce individual cell death in *E. coli* during early phases of phage P1 infection (before new viral particles are produced and released) to prevent destruction of the overall bacterial population (Hazan and Engelberg-Kulka, 2004). When we consider a biofilm, a well-structured and “intelligent” microbial community, this behavior of programmed and altruistic cell death is related to the so-called sacrificial cells: the presence of this phage-derived module ensures that these cells set up an autolytic strategy to supply nutrients to the rest of the population. A second type of behavior frequent in a biofilm is persistence or dormancy: the bacterial cells are slow growing or in steady-state to prevent virus entry (Thingstad et al., 2015) or to counteract nutrient starvation. Even in this case the TA modules play a central role (Lewis, 2012). Therefore, it seems evident that from one side biofilms ensure perfect ecological niche for prophage maintenance and evolution and from the other side phages seem to have contributed to bacterial evolution toward a community lifestyle.

An interesting and peculiar mechanism recently discovered, i.e., explosive cell lysis (Turnbull et al., 2016) underlines the role played by prophages in the formation and release of bacterial MVs, previously described both in Gram-negative (Schwechheimer and Kuehn, 2015) and in Gram-positive (Lee et al., 2009) bacteria. These MVs (50–800 nm, average size 250 nm) can contain different cargo-molecules such as viral genomes (Gaudin et al., 2014), cytoplasmic MPs (Henderson and Martin, 2014), DNA, RNA (Zavan et al., 2020), and are involved in important functions, e.g., bacterial communication through hydrophobic QS signals, HGT, sharing of nutrients (especially evident in the biofilm) (Toyofuku et al., 2017b, 2020), also protecting the cells from some antibiotics and phages by acting as decoys (Manning and Kuehn, 2011).

In *Pseudomonas aeruginosa* a bacteriophage-associated endolysin, encoded into the pyocin gene cluster and coding for a peptidoglycan hydrolase, triggers explosive cell lysis resulting in MVs biogenesis by curling and self-annealing of shattered membrane fragments. Although the pyocin structural genes are not involved, peptidoglycan hydrolase seems not to be the only enzyme responsible for lysis since, at least in *E. coli*, also holins (facilitating translocation of cell wall hydrolases across the inner membrane) participate to the phenomenon. The inductors of the lytic cycle and vesicle formation can be multiple: light, mutagens, antibiotics and other exogenous stressors, however, endogenous events cannot be excluded (Turnbull et al., 2016).

The described model is not the only one acting on the basis of a phage-mediated stimulus. Toyofuku et al. (2017b) reported that prophages can also induce MVs formation on Gram-positive bacteria, although with a different strategy not implying explosive cell lysis. In *B. subtilis* a holin-endolysin mediated mechanism occurs. First, holin causes the formation of pores in the cytoplasmic membrane thus facilitating endolysin hydrolysis of peptidoglycan. Once achieved the disruption of peptidoglycan, the cytoplasmic membrane is extruded and the vesicles released, while the bacterial cell dies because membrane integrity is lost. Phage-related proteins have been demonstrated within the vesicles by proteomic studies in both *B. subtilis* (Kim et al., 2016) and *Streptococcus pneumoniae* (Resch et al., 2016).

When considering both mechanisms, it is interesting to highlight that only a part of the overall population dies: in *P. aeruginosa* not all cells harbor the pyocin-prophage insert and in the Gram-positive model the damage caused to the cytoplasmic membrane cannot always be lethal (Toyofuku et al., 2017a). In general, it can be stated that the generation of vesicles is beneficial not exactly for the individual cell but for the microbial community (e.g., the biofilm) in which nutrient exchange and HGT are favored. Moreover, MVs being similar to intact cells and bearing phage receptors can act as decoys attracting antibiotics and phages, thus ensuring an escape lane to living cells (Manning and Kuehn, 2011). Furthermore, also phages can beneficiate of some advantages: since MVs harbor phage receptors, when they fuse with membranes of phage resistant bacterial cells these not-target bacteria become sensitive to phage attack thus allowing the phage population extending the number of hosts and increasing their diffusion among bacterial species. Therefore, in a circular way, also bacterial communities can beneficiate of further HGT by phage transduction. This ultimately results in enhanced fitness of microbial populations as suggested by Nanda et al. (2015). Finally, it is tempting to speculate that MVs can be one of the possible structures giving origin to the lipid envelope present in some phage families. Curiously, the dsRNA phage phi6 (belonging to the *Cystoviridae* family of rare enveloped phages) displaying a lytic behavior on the target Gram-negative *P. aeruginosa* acquires the envelope from the bacterial cytoplasmic membrane inside the host cytoplasm (Laurinavičius et al., 2004). Experiments, recently reported by Lyytinen et al. (2019), highlight that the phage-encoded protein P9 can induce in *E. coli* the formation of intracellular MVs where the protein (possessing a transmembrane domain) is partially incorporated. The presence of internal organelles in prokaryotes are a very rare event observed in Cyanobacteria (thylakoids) and magnetotactic bacteria only. Here again, although the advantages for the host of such a system are still to be fully elucidated, the interaction between bacteria and viruses can give rise to novel opportunities of evolution.

## Bacteria–Phage–Host: A Russian-Doll Model for an Inter-Kingdom Coordinated Project

**FIGURE 5:**
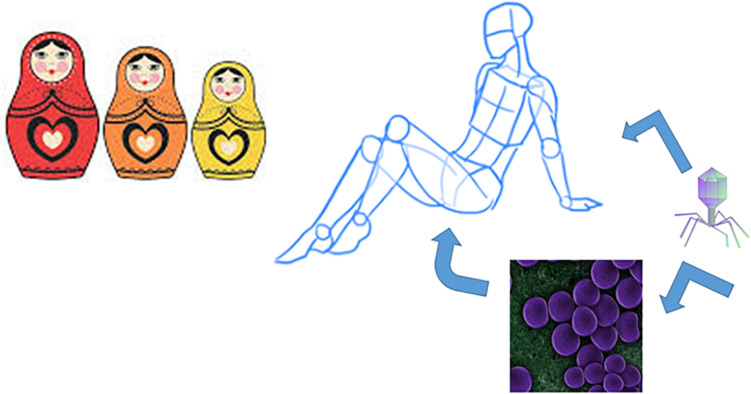
Russian doll model for the multilevel relationships phage–bacteria–human host.

The complex multilevel communication occurring among bacteria, phages and their animal host have driven evolutionary strategies that are worth to be examined. A huge number of inter-kingdom interactions exists in the complex ecosystem of the human gut where many actors are playing on the stage, including viruses, bacteria, archaea, protozoa, and fungi. However, a significant degree of complexity can be observed even when limiting our attention to the central role of bacteria that cross-talk with their inner (phage) and outer (human) host(s), in a Russian doll model represented in Figure 5. First there are some analogies in the behavior that phages and bacteria entertain with their respective host. Similar features characterize phages and bacteria in their ability to set up a form of dormancy (pseudolysogeny in phages and persister cells in bacteria) that allows them waiting for better conditions to activate their reproductive cycle (Łoś and Węgrzyn, 2012). A second analogy is that phage infection is dependent upon the metabolic state of the bacterial-host (De Sordi et al., 2019b) as well as bacterial colonization is dependent upon host metabolism, nutrient availability and circadian rhythms (Rosselot et al., 2016). For instance, planktonic actively duplicating bacterial cells allow to phages high infection efficiency, whereas biofilm-embedded cells are protected from phage (Vidakovic et al., 2018) and viral particle assembly is slow-down during stationary phase (Bryan et al., 2016). In addition, the environment in which bacteria live favors or hinders phage establishment (van Belleghem et al., 2019) like the environment in which the human host lives (hormones, diet, drugs, pollutants, etc…) can favor or exclude certain bacterial populations. Although the mucus layer can harbor a reservoir of bacteria that is maintained thanks to the mucin-derived glycans, regardless of food intake, the lumen microbiota is deeply affected by diet (Donaldson et al., 2016). Actually, the crucial role of diet in selecting saccharolytic or proteolytic bacterial populations has long been established (Gibson and Roberfroid, 1995). Similarly, certain host-environmental conditions can induce production of capsules that mask phage receptors on the bacterial surface of certain species compelling phages to find another target (Roach et al., 2013). Even in this case, however, the arms race between bacteria and viruses favors the appearance of mutant phages that possess enzymes hydrolyzing capsules and ensuring phage access to the receptor (Samson et al., 2013).

On the other hand, it is worth underlining that this multilevel relationship deeply affects the fitness of the three partners finally resulting in a dynamic fluctuation generally driven toward homeostasis (Chaturongakul and Ounjai, 2014), although some possible drifts can occur. It has been experimentally detected that healthy humans display a higher bacterial biodiversity, whereas the phage diversity is increased in inflammatory bowel disease (Norman et al., 2014). Nevertheless, filtered fecal transplantation, although lacking bacterial cells, provides benefits in overcoming *Clostridium difficile* colitis, suggesting that bacteriophages play a very important role in the human gut homeostasis (Ott et al., 2017).

### Partnership Agreement Between Bacteria and the Human Host

The partnership agreement between bacteria and human host to control several aspects of reciprocal well-being is a well-established concern that also includes bacteriophage control. Actually, humans and other animals have evolved a sophisticated immune system not only toward pathogenic bacteria but against viruses as well. Since host immune system does not discriminate between human-targeting viruses and phages, it is likely that immune defenses can control phage populations thus protecting commensal bacteria. At this purpose, it has been demonstrated that viral nucleic acids are recognized by specific toll-like receptors (TLR) namely DNA by TLR9, dsRNA by TLR3 and ssRNA by TLR7 and TLR8, that after sensing the nucleic acid, trigger production of IFNs and other pro-inflammatory cytokines (De Paepe et al., 2014). More specifically, phages have been demonstrated to interact with the animal immune system, by means of head proteins, inducing immunological responses, even if sometimes this interaction can result in a negative modulation of both humoral and cell-mediated immunity probably inducing tolerance toward phages and their bacterial hosts (Da̧browska et al., 2006).

### Partnership Agreement Between Bacteria and Phages

In a second model, bacteria and phages can set up a partnership agreement that ensures phage survival and better bacterial fitness toward the mammalian host. The well-known lysogenic conversion support bacteria in producing toxic molecules (such as cholera, diphtheria, and Shiga toxins) that protect them from the host immune reaction and this also ensures temperate phage stabilization into the bacterial genome (Fortier and Sekulovic, 2013). The severe epidemic outbreak of Shiga-toxin producing *E. coli* O104:H4 occurred in 2011 in Germany was linked to a toxin-encoding phage (Muniesa et al., 2012). An additional example is *P. aeruginosa* PAO1 bearing a prophage, which although less fitting in swimming, swarming and twitching motility, enhances production of the virulence factor pyocianin and increases resistance to macrophages internalization (Hosseinidoust et al., 2013). In the same bacterial model, the phage-like type VI secretion system allows bacterial translocation of virulence factors (Basler et al., 2012). However, virulence factors like toxins can sometimes have also a beneficial action on the animal host such as carcinogenesis suppression as reported by Doulberis et al. (2015).

Besides helping to produce virulence factors, prophages can also modify surface antigenicity of bacteria thus favoring their host in evading the mammalian immune system (De Paepe et al., 2014). In Gram-negative bacteria, modifications in the O-antigen domain of the outer membrane have been described (Davies et al., 2013). A curious example of surface modification involving a three-partner interaction, has been reported by Mitchell et al. (2007): in this case prophage induction occurs and the production of holins provides enhanced cell permeability and secretion of some parts of the phage “body,” namely, tail fiber proteins that are exposed on the bacterial (*Streptococcus mitis*) cell surface of not fully lysed cells where they act as adhesins for platelet cells. Furthermore, there is growing evidence that harboring a prophage, besides enhancing aggressiveness toward the host, is a winning strategy for bacteria to improve their resilience to harsh conditions such as presence of bile salts and oxidants, not so rare in the human gut (Wang et al., 2010).

In all the reported examples, phage-bacterial host relationship can be viewed as mutualistic as suggested by Beckett and Williams (2013). Actually, phages provide competitiveness to their bacterial-host in a way that probably proved useless in an abiotic ecological niche (where lysogeny is less observed, see above). On its side, the bacterium continues to cultivate this successful interaction by harboring the virus as a prophage to defend itself from the mammalian host. Therefore, the human/animal host represents a crucial factor for maintaining phage lysogeny. On the other hand, this has been demonstrated also by the abundance of integrase genes detected by metagenomics in the genome of phages living in the gut community (Minot et al., 2011).

### Partnership Agreement Between Phages and the Human Host

A third scenario is possible in which the partnership agreement can be signed also between phages and human host. Phages are the most represented entities in the biosphere. It has been established that the phage population in the human gut (where the number of phages reaches 10^9^ per gram versus 10^11^ bacteria) (Kim et al., 2011) is higher than that of human-targeting viruses (De Paepe et al., 2014). However, it is difficult to establish which families of viruses are present because of the lack of molecular genomic markers similar to the bacterial 16S ribosomal RNA genes, but also because extracting the genetic content from viral particles is still challenging (Roux et al., 2013). According to Manrique et al. (2017) the most represented families are the dsDNA Myoviridae, Siphoviridae and Podoviride and also the ss DNA Microviridae that represent the healthy gut phageome. In some cases, phages, due to their killing activity (lytic cycle) on bacteria can behave as antimicrobial agents controlling bacterial populations and preventing bacterial infection: this has been related to the high number of bacteriophages present at the gut level on mucosal surfaces and interacting with the mucus layer by means of an immunoglobulin-like capsid domain (Barr et al., 2013). According to these authors, host mucosal surfaces and phage coevolve to maintain phage adherence. From one side, this benefits the host since phage adherence to mucus provides a non–host-derived antimicrobial defense, limiting the number of mucosal bacteria and contributing to the maintenance of a selected commensal microbiota. Actually, based on the model kill-the-winner (De Paepe et al., 2014), phage can especially control excessive growth of a specific bacterial population, as occurs during a sudden invasion by an exogenous pathogen, but also when dysbiosis favors a particular endogenous lineage. On the other side, it offers to the phage the opportunity to encounter bacteria thus potentially increasing their replicative success (Barr et al., 2013). Alongside this model that represents a true metazoan-phage symbiosis, indirect effects positively affecting host fitness are reported: phage head and tail proteins can stimulate the immune system sometimes inducing antibodies and pro-inflammatory cytokines (Łusiak-Szelachowska et al., 2014) sometimes reducing inflammation caused by bacterial LPS (Miernikiewicz et al., 2016). A recent and very interesting article suggests that dietary fructose and bacterial-derived SCFA can trigger phage transition from lysogenic to lytic (Oh et al., 2019). This is not the only example in which human host-derived molecules such as component of the western diet, food additives ad nicotine can affect phage populations by controlling their bacterial hosts (Mirzaei and Maurice, 2017). Furthermore, sub-inhibitory doses of antibiotics can trigger prophage excision from bacterial DNA and lytic cycle establishment, thus enhancing bacterial killing, both in *E. coli* (Zhang et al., 2000) and in pathogenic Gram-positive models resulting in phage expansion and bacterial infection control (Matos et al., 2013). However, it has been reported that most stressors (oxidants, smoke, and antibiotics) mainly induce prophage lytic cycle on beneficial microbes rather than on opportunistic pathogens thus triggering gut dysbiosis (Mills et al., 2013). Therefore, it seems reasonable to assume that the partnership agreement to be beneficial should occur in specific physiological conditions.

### Three-Partner Agreement

Finally, it is of interest considering a three-partner agreement: the one mediated by the relatively recently discovered MVs. As discussed in a previous section, MVs exist because of an integrated prophage into a bacterial genome. Besides being important for phage propagation and bacteria–bacteria interaction, MVs can also play a role in the mammalian host physiology, in pathological states and in the reciprocal interactions occurring between host and bacteria. Actually, it has been demonstrated that MVs release can be induced by animal host signals such as changes in temperature, pH, presence of antimicrobial peptides (Deatherage and Cookson, 2012).

As far as the control of host physiology is concerned, MVs can stimulate protective immunity controlling both innate and adaptive immune responses (Deatherage and Cookson, 2012) and also regulate inflammatory pathways (Alaniz et al., 2007). Moreover, MVs from *Listeria monocytogenes* can inhibit autophagy (Vdovikova et al., 2017) and it has been reported that MVs from *Akkermansia muciniphyla* (but not those from *E. coli*) can regulate tight junctions thus controlling intestinal barrier integrity and gut permeability with consequences on the overall inflammatory state of the host organism (Chelakkot et al., 2018).

Modulation of pathological states of the host, such as cancer, by MVs has also been described. Vdovikova et al. (2018) recently demonstrated that MVs from pathogenic (but not from commensal) bacteria such as *Vibrio cholerae*, can impact gene expression on colon carcinoma cells, by enhancing the transcription of genes involved in cell differentiation. This effect, probably involving epigenetic changes and chromatin accessibility, do not require direct contact between bacteria and cancer cells (Vdovikova et al., 2018). If other cellular structures are involved in the interaction with the bacterial MVs remains to be elucidated. Furthermore, MVs from *V. cholerae* display the ability to induce higher expression of the nuclear receptor for the Vitamin D that promotes differentiation of colon carcinoma cells (Pálmer et al., 2001).

It is evident from all these examples that the phenotypic effect, finally resulting after MVs signaling to the host, strongly depends upon the cargo transported by the MV (toxin, immunomodulating molecule, and non-human-targeted compound). The contact between MV and the host cell can occur either by endocytosis or by membrane fusion (O’Donoghue and Krachler, 2016) however, in both cases this strategy potently enhances the efficiency by which the active compound (transported and protected) is sensed by the target cell.

Membrane vesicles can also shape the pathogen–host interaction, sometimes favoring sometimes attenuating pathogenesis. Two examples are worth mentioning. In the *Salmonella enterica* serovar Thypy model, the Cly A cytotoxin is eight fold more active in causing host cell lysis when embedded in MVs than in the free form. The fusion of MVs with the cell membrane of the eukaryotic host can facilitate the toxic action (Wai et al., 2003). On the contrary, in the *Helicobacter pylori* model the MVs-associated toxin VacA is less active than the soluble form, suggesting that the use of MVs is a strategy used by bacteria to modulate the virulence impact during infection (Ricci et al., 2005). This ambiguity still remains considering the fact that MVs from one side can act as decoys attracting the antibody response, thus allowing the producing bacterium to escape the host immune system, from the other side, they can represent a first signal that allows early detection of the bacterial population by the host immunity that can thus organize and react accordingly (Deatherage and Cookson, 2012). All these data underline that the complex interplay between phages, bacteria and the host is far to be fully understood.

## Conclusion

From the analysis of the intricate relationships established between bacteria and their inner and outer hosts, two essential aspects emerge: i) bacteria carry out strategies finalized to communication but also to earn energy and make the cheapest choice, ii) bacteria integrate the “enemy” in view of a broader advantage on the long period and in a larger context. Both behaviors are sustainable. From one hand, they take into account the possibility to use simple building blocks to perform multiple duties (i.e., multitasking signaling compounds and MPs), sometimes using module differentiation (PTMs). From the other hand, the logic of sharing and reuse, together with the conflict mediation has brought during evolution to a shift from a negative event (phage attack) into big opportunities of survival in systemic communities, which ultimately promote a more complex level of organization such as social life and the establishment of a successful integrated lifestyle.

The overall reported data of the last section highlight that a holistic viewpoint can better gave us a framework of what really happens in the phage–bacteria–animal host multilevel community. Phages are the software of bacteria like symbiont bacteria are the software of humans, in a Russian doll-like model. Bacteria are not the same without their prophage(s) and humans are not the same without their tailor-made microbiota. Both can be removed from their respective hosts, however, the hosts pay a cost in reducing its fitness (less resistant/performant bacteria and dysbiosis). Despite their bad reputation due to the fact that both phage and bacteria can kill their host, phages can provide bacteria with new genes, supplying them new opportunities and commensal bacteria can offer to the human hosts new metabolic and signaling pathways not supported by host genome. An agreement often occurs to ensure that the war can be changed into a reciprocally profitable event. Every level sustains intricate and complex relationships with the other levels (and other gut populating organisms as well) and, although more than expected interactions also occur between phages and human host, bacteria are in the center. In this sometimes intricate network of possibilities they prove to be efficient, intelligent in decision-making, and able to respond to both sides. A good lesson also for humans.

## Author Contributions

The author confirms being the sole contributor of this work and has approved it for publication.

## Conflict of Interest

The authors declare that the research was conducted in the absence of any commercial or financial relationships that could be construed as a potential conflict of interest.
